# Supplementary material for: Cocaprins, β-Trefoil Fold Inhibitors of Cysteine and Aspartic Proteases from Coprinopsis cinerea
Source: Int J Mol Sci. 2022 Apr 28;23(9):4916. doi: 10.3390/ijms23094916 (PMC9104457; doi:10.3390/ijms23094916)
Supplement: Supplementary file 1 [file ijms-23-04916-s001.zip › ijms-1693461-supplementary.pdf]

International Journal of *Molecular Sciences*

MDPI

SUPPLEMENTARY MATERIALS

Cocaprins, β-trefoil fold inhibitors of cysteine and aspartic proteases from *Coprinopsis cinerea*

Miha Renko, Tanja Zupan, David F. Plaza, Stefanie S. Schmieder, Milica Perišić Nanut, Janko Kos, Dušan Turk, Markus Künzler and Jerica Sabotič

Supplementary Table S1. Ranked mammalian printed glycan array data of CCP1. Glycans are ranked according to the measured RFU (relative fluorescence units) and only results with RFU above 1000 are shown. High concentrations of CCP1 and CCP2 (200 μg/ml) were used in the analyses, in which case nonspecific binding is possible. For CCP2, no binding was observed above the cut-off. See Figure S5 for graphical representation.

Chart Number	Glycan-spacer	AvgMeanS-B w/o MIN/MAX	StDev	%CV
540	Galb1-4GlcNAcb1-3Galb1-4GlcNAcb1-2Mana1-6(Galb1-4GlcNAcb1-3Galb1-4GlcNAcb1-2Mana1-3)Manb1-4GlcNAcb1-4GlcNAcb-Sp12	2395	491	20
582	GlcNAcb1-3Galb1-4GlcNAcb1-3Galb1-4GlcNAcb1-6(GlcNAcb1-3Galb1-4Glc-NAcb1-3Galb1-4GlcNAb1-2)Mana1-6(GlcNAcb1-3Galb1-4GlcNAcb1-3Galb1-4GlcNAcb1-2Mana1-3)Manb1-4GlcNAcb1-4(Fuca1-6)GlcNAcb-Sp24	2000	350	17
584	GlcNAcb1-3Galb1-4GlcNAcb1-3Galb1-4GlcNAcb1-3Galb1-4GlcNAcb1-6(Glc-NAcb1-3Galb1-4GlcNAcb1-3Galb1-4GlcNAcb1-3Galb1-4GlcNAb1-2)Mana1-6(GlcNAcb1-3Galb1-4GlcNAcb1-3Galb1-4GlcNAcb1-3Galb1-4GlcNAcb1-2Mana1-3)Manb1-4GlcNAcb1-4(Fuca1-6)GlcNAcb-Sp24	1834	132	7
544	GlcNAcb1-3Galb1-4GlcNAcb1-3Galb1-4GlcNAcb1-2Mana1-6(GlcNAcb1-3Galb1-4GlcNAcb1-3Galb1-4GlcNAcb1-2Mana1-3)Manb1-4GlcNAcb1-4GlcNAcb-Sp12	1633	434	27
567	Galb1-4GlcNAcb1-3Galb1-4GlcNAcb1-3Galb1-4GlcNAcb1-3Galb1-4GlcNAcb1-3Galb1-4GlcNAcb1-3Galb1-4GlcNAcb1-2Mana1-6(Galb1-4GlcNAcb1-3Galb1-4GlcNAcb1-3Galb1-4GlcNAcb1-3Galb1-4GlcNAcb1-3Galb1-4GlcNAcb1-3Galb1-4GlcNAcb1-2Mana1-3)Manb1-4GlcNAcb1-4GlcNAcb-Sp25	1375	227	17
546	Galb1-4GlcNAcb1-3Galb1-4GlcNAcb1-3Galb1-4GlcNAcb1-2Mana1-6(Galb1-4GlcNAcb1-3Galb1-4GlcNAcb1-3Galb1-4GlcNAcb1-2Mana1-3)Manb1-4Glc-NAcb1-4GlcNAcb-Sp12	1371	337	25
557	GlcNAcb1-3Galb1-4GlcNAcb1-6(GlcNAcb1-3Galb1-4GlcNAcb1-2)Mana1-6(Glc-NAcb1-3Galb1-4GlcNAcb1-2Man a1-3)Manb1-4GlcNAcb1-4GlcNAc-Sp24	1303	45	3
577	GlcNAcb1-3Galb1-4GlcNAcb1-3Galb1-4GlcNAcb1-3Galb1-4GlcNAcb1-2Mana1-6(GlcNAcb1-3Galb1-4GlcNAcb1-3Galb1-4GlcNAcb1-3Galb1-4GlcNAcb1-2Mana1-3)Manb1-4GlcNAcb1-4(Fuca1-6)GlcNAcb-Sp24	1258	158	13

605	Neu5Aca2-6Galb1-4GlcNAcb1-3Galb1-4GlcNAcb1-3Galb1-4GlcNAcb1-2Mana1-6(Neu5Aca2-6Galb1-4GlcNAcb1-3Galb1-4GlcNAcb1-3Galb1-4GlcNAcb1-2Mana1-3)Manb1-4GlcNAcb1-4GlcNAcb-Sp12	1256	628	50
587	Galb1-4GlcNAcb1-3Galb1-4GlcNAcb1-3Galb1-4GlcNAcb1-3Galb1-4GlcNAcb1-3Galb1-4GlcNAcb1-6(Galb1-4GlcNAcb1-3Galb1-4GlcNAcb1-3Galb1-4GlcNAcb1-3Galb1-4GlcNAcb1-3Galb1-4GlcNAb1-2)Mana1-6(Galb1-4GlcNAcb1-3Galb1-4GlcNAcb1-3Galb1-4GlcNAcb1-3Galb1-4GlcNAcb1-3Galb1-4GlcNAcb1-2Mana1-3)Manb1-4GlcNAcb1-4(Fuca1-6)GlcNAcb-Sp24	1090	143	13
371	Gala1-3Galb1-4(Fuca1-3)GlcNAcb1-2Mana1-6(Gala1-3Galb1-4(Fuca1-3)Glc-NAcb1-2Mana1-3)Manb1-4GlcNAcb1-4GlcNAcb-Sp20	1085	74	7
583	Galb1-4GlcNAcb1-3Galb1-4GlcNAcb1-3Galb1-4GlcNAcb1-6(Galb1-4GlcNAcb1-3Galb1-4GlcNAcb1-3Galb1-4GlcNAb1-2)Mana1-6(Galb1-4GlcNAcb1-3Galb1-4GlcNAcb1-3Galb1-4GlcNAcb1-2Mana1-3)Manb1-4GlcNAcb1-4(Fuca1-6)Glc-NAcb-Sp24	1019	82	8
606	Neu5Aca2-3Galb1-4GlcNAcb1-3Galb1-4GlcNAcb1-3Galb1-4GlcNAcb1-2Mana1-6(Neu5Aca2-3Galb1-4GlcNAcb1-3Galb1-4GlcNAcb1-3Galb1-4GlcNAcb1-2Mana1-3)Manb1-4GlcNAcb1-4GlcNAcb-Sp12	1013	300	30
581	Galb1-4GlcNAcb1-3Galb1-4GlcNAcb1-6(Galb1-4GlcNAcb1-3Galb1-4GlcNAb1-2)Mana1-6(Galb1-4GlcNAcb1-3Galb1-4GlcNAcb1-2Mana1-3)Manb1-4Glc-NAcb1-4(Fuca1-6)GlcNAcb-Sp24	1000	102	10

Supplementary Table S2: List of oligonucleotides used in cocaprin cloning and PCR site-directed mutagenesis. Introduced restriction enzyme sites and His-tag are underlined and altered bases in mutagenic oligonucleotides are indicated in bold type.

**Primer name**	**Sequence 5' → 3'**
CC1G_05299 Fw8HisNdeI	ggggcatatgcatcatcatcatcatcatcaccacgagcctggacgatacag
CC1G_05299 RvNotI	cgcggccgctcaagcctcttcaacaaccc
CC1G_05298 FwNdeI	gtacagcatatggttgaacccggacgatac
CC1G_05298 Rv8HisBamHI	gcgcccggatccttaatgatgatgatgatgatgatgatgcataccagcctcaaag
CCP1_ G13E_f	gtcattaatgtcaag**GAG**ggcacagcccttgac
CCP1_ G13E_r	gtcaagggctgtgcc**CTC**cttgacattaatgac
CCP1_N22R_f	cttgacctcgatatt**CGC**aacaactcaactgttc
CCP1_N22R_r	gaacagttgagttgtt**GCG**aatatcgaggtcaag
CCP1_ FH32EE_f	gttcatggatgggcc**GAAGAG**ggcggcgacaacc
CCP1_ FH32EE_r	ggttgtcgccgcc**CTCTTC**ggcccatccatgaac
CCP1_D47R_f	ctttgagcacatagga**CGT**aacatctggactatat
CCP1_D47R_r	atatagtccagatgtt**ACG**tcctatgtgctcaaag

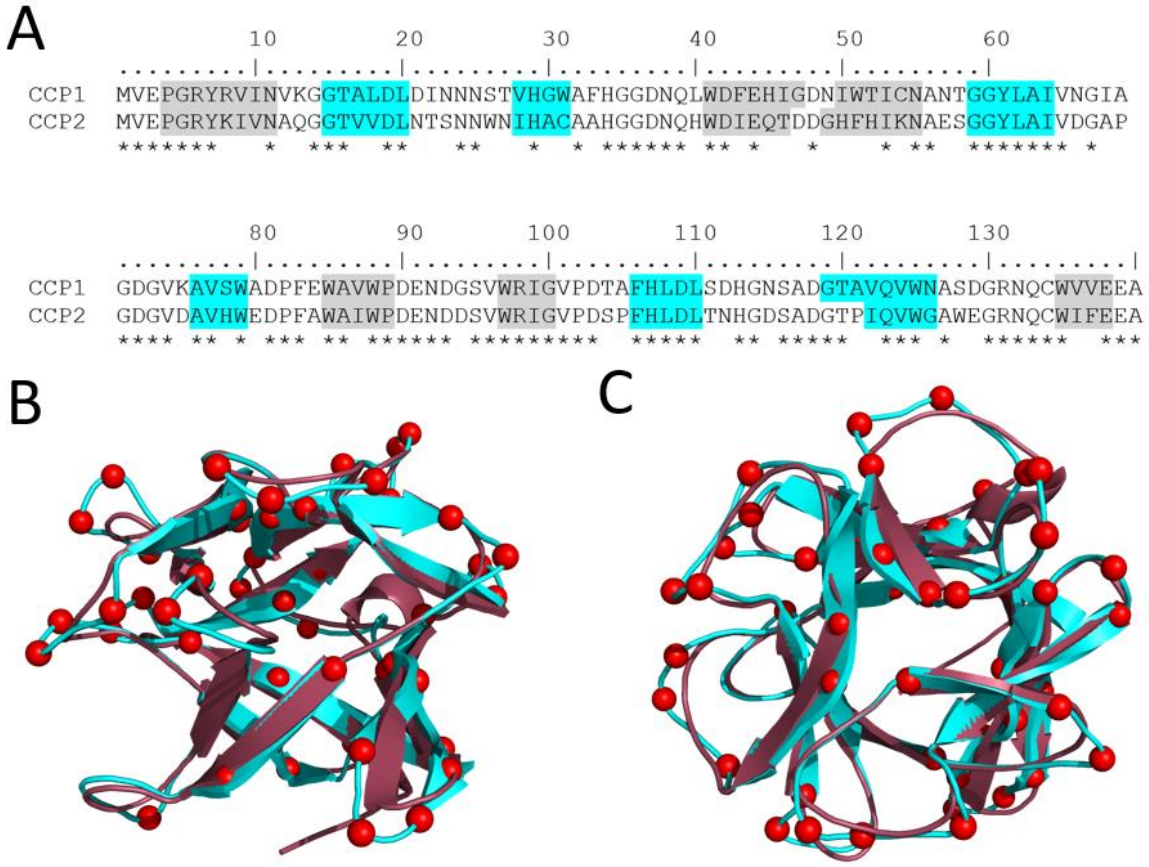

Supplementary Figure S1. **Comparison of CCP1 and CCP2.** A) Sequence alignment of CCP1 and CCP2. β-strands are shown in gray and cyan for β-strands in the trunk and crown regions, respectively. Asterisks below the alignment indicate conserved residues. B) and C) Structural alignment of the CCP1 crystal structure (cyan) and the CCP2 AlphaFold2 model (dark red) in two different orientations. The red spheres represent the residues that are different between the two proteins.

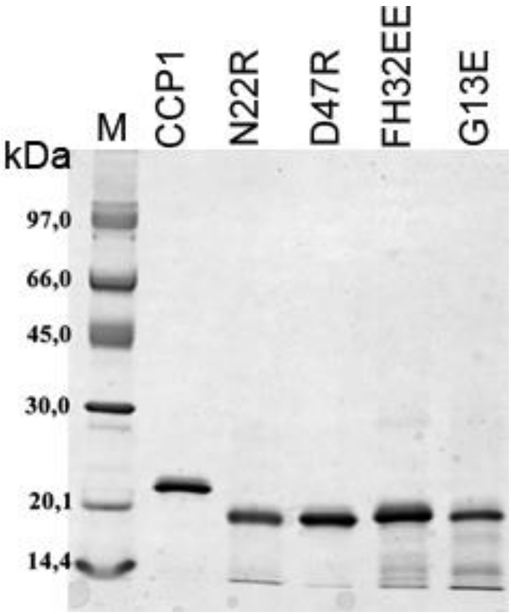

Supplementary Figure S2. SDS-PAGE analysis of CCP1 and its mutants in a 15% polyacrylamide gel under denaturing conditions and Coomassie blue staining.

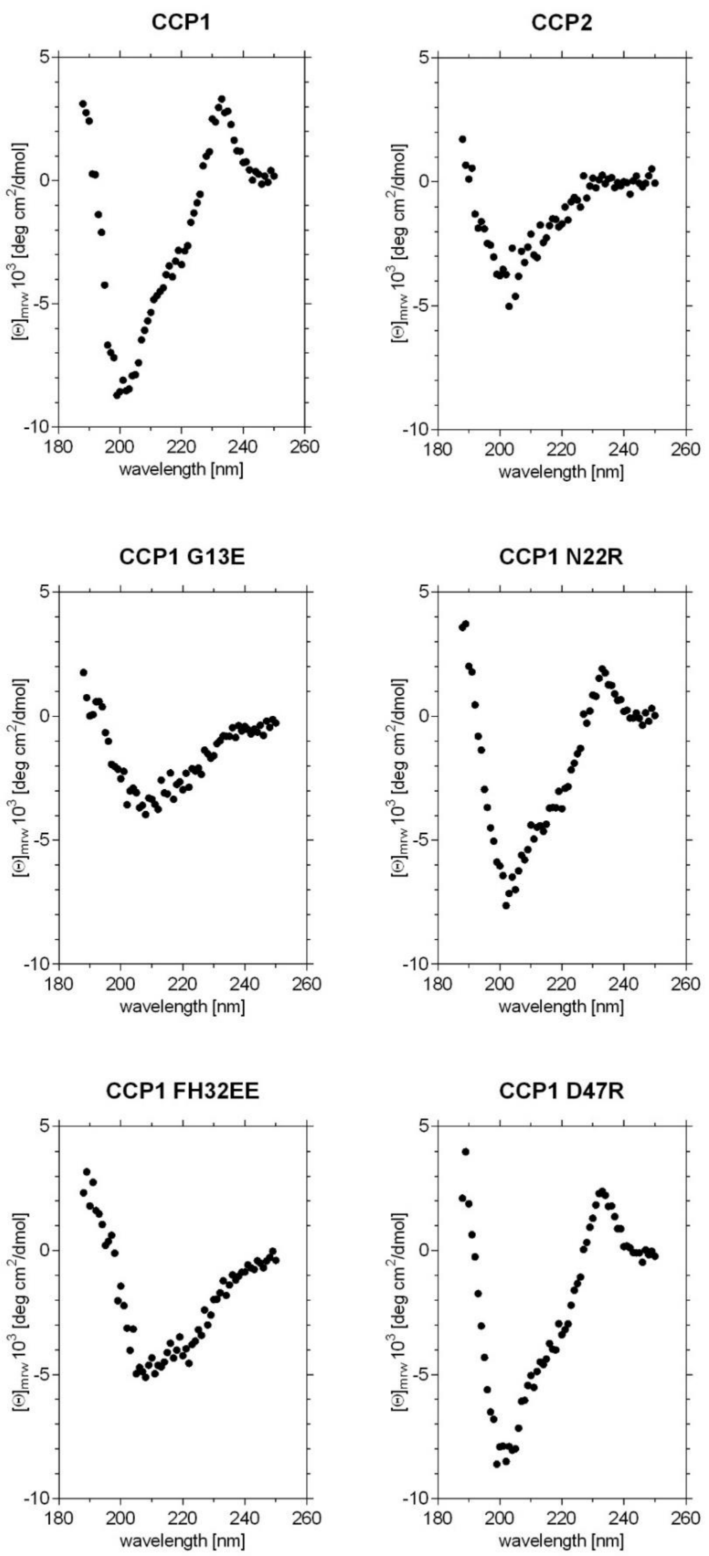

Supplementary Figure S3. CD spectra of CCP1, CCP2 and different CCP1 mutants. CD spectra were recorded from 185–250 nm for proteins (0.2 mg/ml) in 10 mM phosphate buffer pH 6.5 on an Chirascan (Applied Photophysics) spectropolarimeter using 1 mm cell (HellmaAnalytics). Buffer lines were subtracted from corresponding spectra and the data transformed to mean residue elipticity.

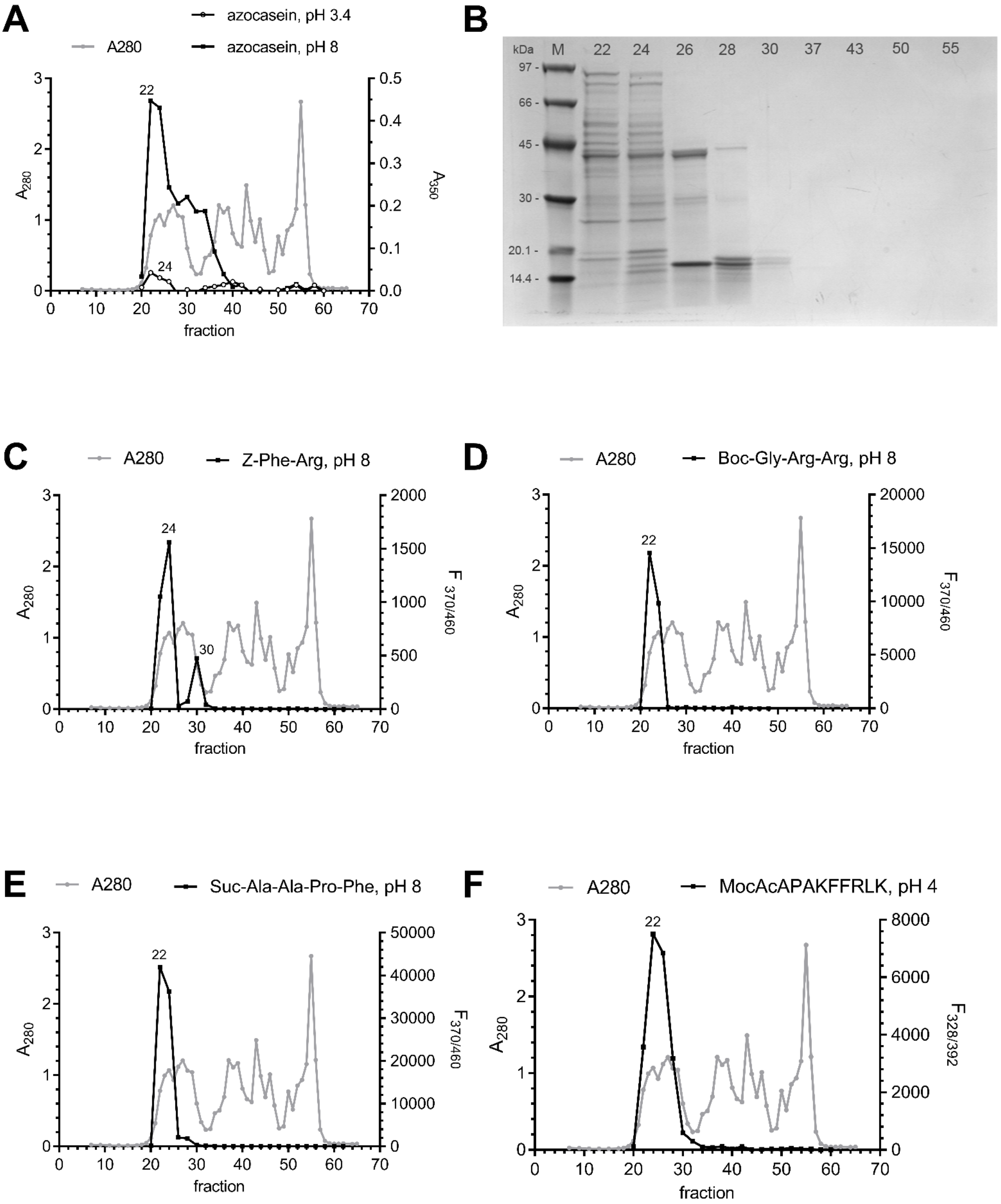

Supplementary Figure S4. Proteolytic activities in gel filtration fractions of *C. cinerea* fruiting body extract. Crude protein extract was prepared from lyophilized immature fruiting bodies (1.3 g extracted in 60 ml 20 mM Tris-HCl buffer

with 0.3M NaCl, pH 7.5). Undissolved material was removed by centrifugation and the extract concentrated by ultrafiltration (3 kDa cut-off) to 4 ml. The extract was applied to an analytical gel filtration column and fractionated on Sephacryl S100 (19.2 ml/h, 6 ml fractions collected). Absorbance at 280 nm in fractions is shown in gray in panels A, C, D, E, F and SDS-PAGE analysis of selected fractions in an 12 % polyacrylamide stained in Commasie blue is shown in panel B. Proteolytic activity was assayed in gel-filtration fractions (black lines in panels A, C, D, E, F) against azocasein (Sigma) at pH 3.4 and pH 8, against Z-Phe-Arg-MCA, Boc-Gly-Arg-Arg-MCA and Suc-Ala-Ala-Pro-Phe-MCA (all from Bachem) in Tris-buffer at pH 8 and against MOCAc-Ala-Pro-Ala-Lys-Phe-Phe-Arg-Leu-Lys-(Dnp)-NH2, Z-Arg-Arg-pNA (Peptide Institute Inc.) in citric acid phosphate buffer at pH 4. The released absorbance or fluorescence was measured using an Infinite®M1000 microplate reader (Tecan). Fraction showing the highest activity is indicated in each panel.

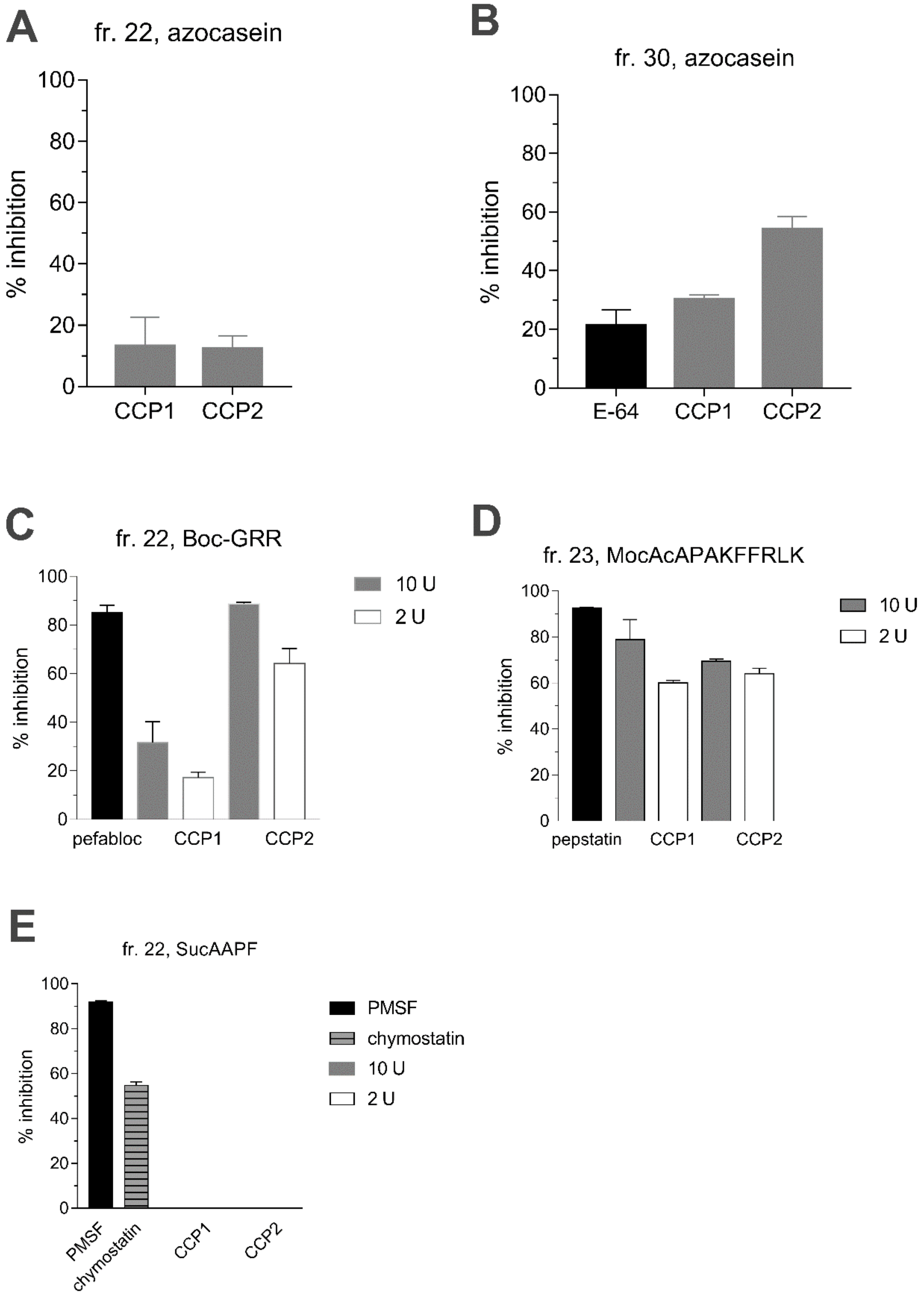

Supplementary Figure S5. Inhibition of proteolytic activities in selected gel filtration fractions of *C. cinerea* fruiting body extract by CCP1 and CCP2 (at two concentrations, indicated as 10 U (gray bars) and 2 U (white bars); u, units). The same gel filtration fractions and conditions were used as in Supplementary Fig. S4. Inhibition of proteolytic activity by CCP1 and CCP2 was assayed in gel-filtration fractions 22 (A) and 30 (B) against azocasein (Sigma) and in fraction 23 (D) against MOCAc-Ala-Pro-Ala-Lys-Phe-Phe-Arg-Leu-Lys-(Dnp)-NH_2 (Peptide Institute Inc.) at pH 4 to demonstrate inhibition of endogenous aspartic proteases. Inhibition of cysteine- and serine-type proteolytic activity by CCP1 and

CCP2 was assayed in gel filtration fraction 22 against Boc-Gly-Arg-Arg-MCA and Suc-Ala-Ala-Pro-Phe-MCA (both from Bachem) in Tris buffer at pH 8. The released absorbance or fluorescence was measured using an Infinite®M1000 microplate reader (Tecan) and then normalized to control without inhibitors to indicate the percentage of inhibition. The protease inhibitors pefabloc (AEBSF; 4-(2-aminoethyl)benzenesulfonylfluoride hydrochloride; 5 mM) (Roche Applied Science), PMSF (phenylmethylsulfonyl fluoride; 1 mM), chymostatin (100 μM) (Sigma), E-64 (l-trans-3-carboxyoxiran-2-carbonyl-l-leucylagmatine; 5 μM) (Peptide Institute Inc.), or pepstatin (6 μM) (Peptide Institute Inc.) were used as controls.

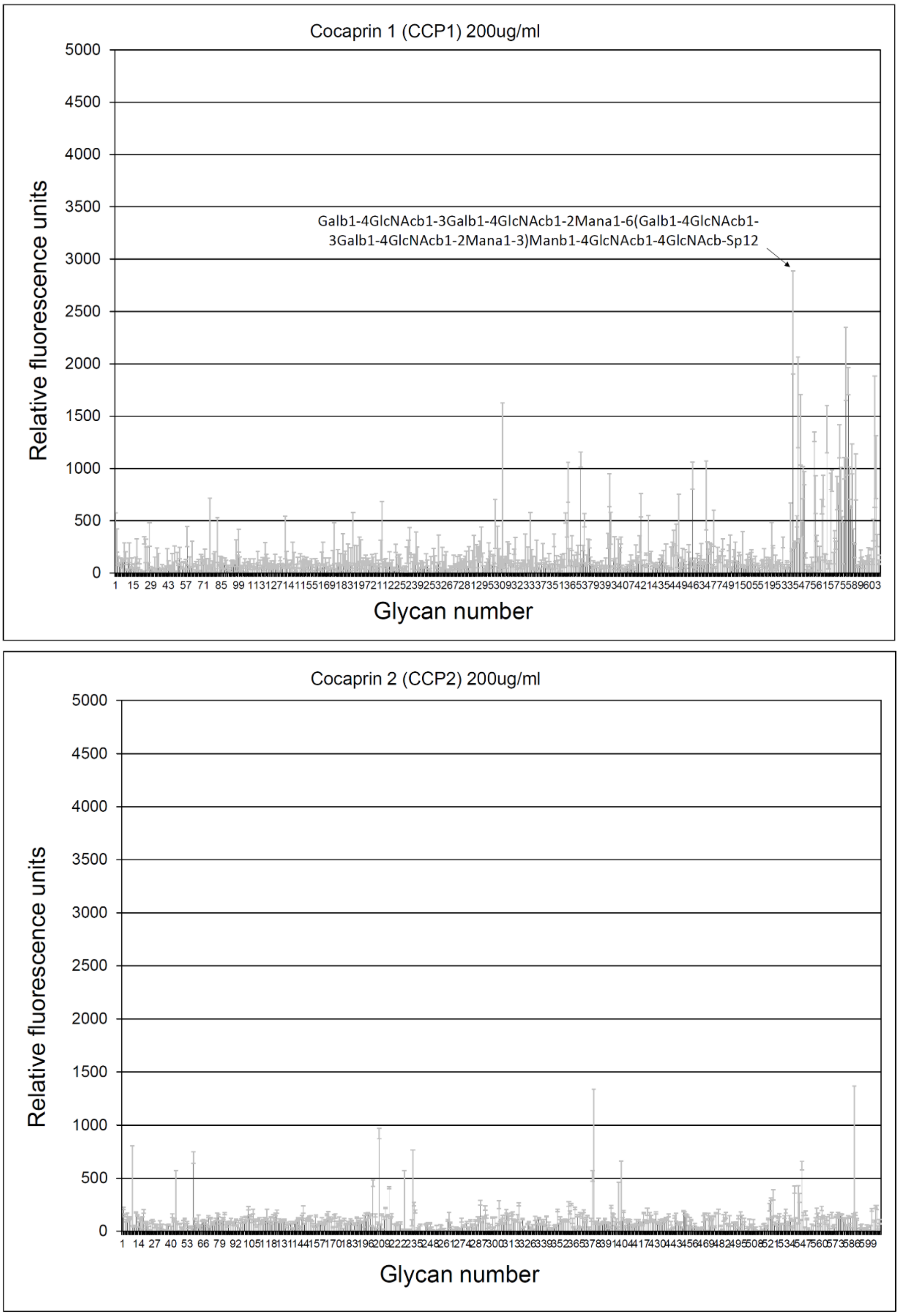

Supplementary Figure S6. Glycan microarray analysis of CCP1 (top) and CCP2 (bottom) with a concentration of 200 μg/ml performed using Mammalian Printed Array version 5.2 of the Consortium for Functional Glycomics. Bars represent relative fluorescence units indicating the specificity of protein for individual glycans and error bars represent standard deviations of the mean fluorescence. The glycan with highest activity on the glycan microarray probed with CCP1 is indicated.

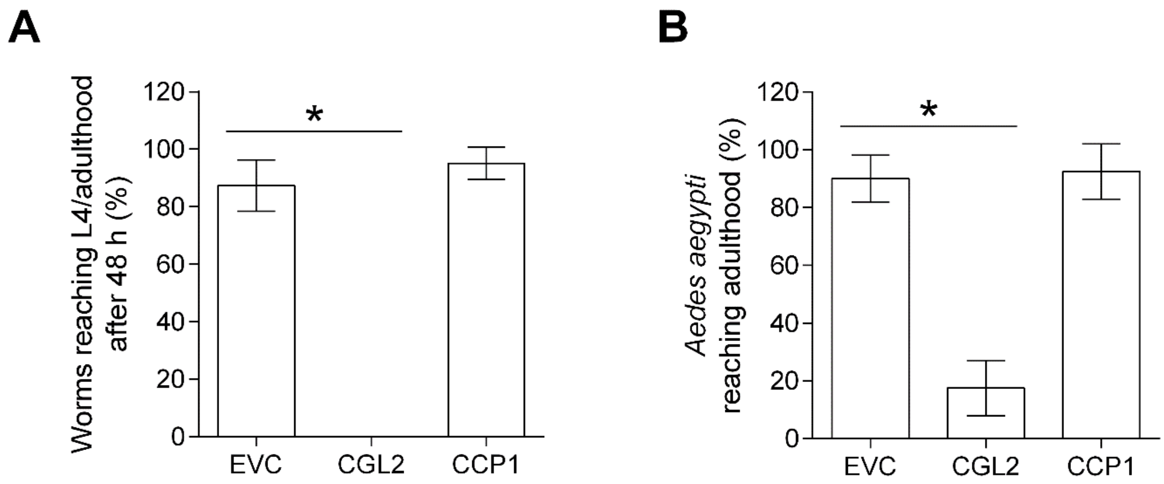

Supplementary Figure S7. CCP1 is not toxic to *C. elegans* or *A. aegypti* larvae. Toxicity of CCP1-expressing *E. coli* towards *C. elegans pmk-1 (km25)* (A) and *A. aegypti* (B) larvae was assessed as previously described [35]. *E. coli* BL21 expressing the toxic lectin CGL2 was used as a positive control, whereas bacteria carrying empty pET24 vector (EVC) were used as a negative control of toxicity. CCP1 was not toxic to insect or nematode larvae. Dunn's multiple comparison test was used to assess the statistical significance of the differences observed between treatments and the negative control. * $0.01 \le$ p-value ≤ 0.05

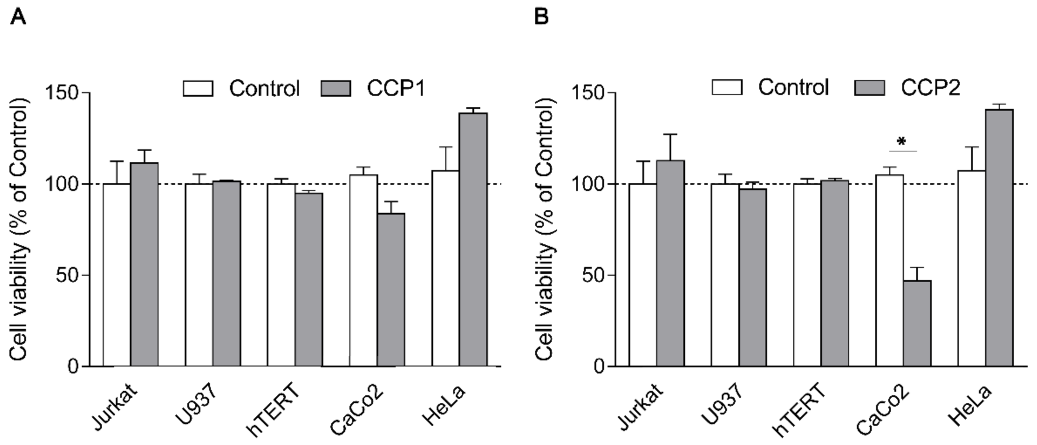

Supplementary Figure S8. The effects of cocaprins on different human cell lines. Viability of different cell lines after 48 hours of incubation with 100 μg/ml of (**A**) cocaprin 1 (CCP1) and (**B**) cocaprin 2 (CCP2) (gray bars). Asterisks above bars indicate statistically significant differences (* $p < 0.05$) between viability of control and cocaprin treated cells. Bars represent means ± SD of the percentage of control cells (white bars), each determined in three independent experiments. The following cell lines were used for cell viability assays: non-differentiated histiocytic lymphoma cells (U937, American Type Culture Collection (ATCC), Manassas, VA, USA, CRL-1593.2), acute T-cell leukemia (Jurkat, ATCC, TIB-152), colorectal adenocarcinoma (CaCo-2, ATCC, HTB-37), cervical cancer cells (HeLa, ATCC, CCL-2), hTert immortalized human microglia, Hμglia (kind gift from Prof. David Alvarez-Carbonell). Cells were grown under ATCC recommended culture conditions or as described [44]. Heat-inactivated FBS and penicillin/streptomycin were from Gibco, USA. Cells were maintained at 37°C in a humidified atmosphere containing 95% air and 5% CO_2. The viability of the various human cell lines after the addition of cocaprins was assessed using the MTS assay CellTiter 96 AQueous One Solution Cell Proliferation Assay (Promega, Madison, WI, USA) according to the manufacturer's instructions. For the MTS cell viability assay, the number of seeded cells/well ($0.7 \times 10^4 – 6 \times 10^4$) varied depending on the cell type used [45]. Recombinant cocaprins (100 μg/ml) were added to cells in 96-well plates, and cell viability was determined after 48 hours. The assay was performed according to the manufacturer's instructions, and absorbance was measured at 490 nm on the Infinite M1000 microplate reader (Tecan). Results are presented as a percentage of the corresponding control cells.
